# Volcano: a pipeline to characterize long terminal repeat-retrotransposons families in plants

**DOI:** 10.1093/bioadv/vbaf162

**Published:** 2025-07-04

**Authors:** Hao He, Fei Shen, Yong Hou, Xiaozeng Yang

**Affiliations:** College of Life Sciences, University of Chinese Academy of Sciences, Beijing 100049, China; Institute of Biotechnology, Beijing Academy of Agriculture and Forestry Sciences, Beijing 100097, China; State Key Laboratory of Plant Diversity and Specialty Crops, Institute of Botany, Chinese Academy of Sciences, Beijing 100093, China; Institute of Biotechnology, Beijing Academy of Agriculture and Forestry Sciences, Beijing 100097, China; National Innovation Center for Digital Seed Industry, Beijing 100097, China; BGI-Shenzhen, Shenzhen 518083, China; State Key Laboratory of Plant Diversity and Specialty Crops, Institute of Botany, Chinese Academy of Sciences, Beijing 100093, China

## Abstract

**Motivation:**

Long Terminal Repeat Retrotransposons (LTR-RTs) comprise a significant portion of repetitive sequences in numerous plant species. LTR-RTs hold considerable functional significance, as they can impact gene family functionality and contribute to the formation of new genes. Investigating the quantities and activities of LTR-RTs is essential for understanding species’ evolutionary dynamics and the foundational mechanisms driving genome evolution. While current softwares can predict and initially classify LTR-RTs, there is a high need for more comprehensive and efficient software to fully characterize and quantify LTR-RTs during burst events and in subsequent detailed classification and quantification, especially given the surged demands of genome annotation.

**Results:**

In this study, we have developed a pipeline called Volcano to accurately classify LTR-RTs and characterize burst families in plants. To distinguish different clades of LTR-RTs, we have implemented an improved depth-first search algorithm. Volcano can also quantify LTR-RT expression using RNA-seq data. By analyzing LTR-RTs in three genomes from the Asteraceae family, we observed that larger genomes tend to contain a greater number of LTR-RTs, and our software effectively categorizes them at the clade level.

**Availability and implementation:**

The proposed Volcano compressor can be downloaded from https://github.com/Suosihe/volcano_LTR.

## 1 Introduction

Transposable elements (TEs) are dynamic components of eukaryotic genomes, playing a crucial role in shaping genome structure, driving evolution and facilitating adaptation (Wessler 2006). TEs possess the unique ability to move and replicate within genomes, significantly enhancing genome plasticity and size variation (Woodford and Johnson 2004). A prominent TE class, Long Terminal Repeat retrotransposons (LTR-RTs), are abundant in plant genomes and contribute significantly to genome size augmentation through their specific copy-and-paste transposition mechanism. They transpose *via* RNA intermediates and are typically flanked by two long terminal repeats (Kumar and Bennetzen 1999). LTR-RTs are significant contributors to genome evolution and biodiversity. For example, retrotransposons make up about three-quarters of the maize (*Zea mays*) genome size (Schnable *et al.* 2009), and in *Oryza australiensis*, LTR-RTs can potentially double the genome size (Piegu *et al.* 2006). In the Asteraceae family, significant genome size fluctuations driven by transposon activity contribute to its high adaptability (Shen *et al.* 2023a,b). In the prevailing epoch of extensive genome assembly, the accurate annotation of LTR-RTs is increasingly important for genome characterization. As vital drivers of genome evolution, LTR-RT s influence the gain and loss of genes, leading to the emergence of novel traits and the formation and evolution of species. The identification of LTR-RTs has played a crucial role in revolutionizing our understanding of genetic dynamics.

The classification of LTR-RTs has long been challenging due to their extensive diversity and high abundance. Based on their unique structural coding domains, LTR-RTs are mainly divided into the Ty1/*Copia* and Ty3/*Gypsy* superfamilies (Wicker *et al.* 2007). Using a similar approach on varying domains within LTR-RTs, research by Carlos Llorens and Pavel Neumann identified that in Magnoliophyta, all Ty1/*Copia* families with conserved reverse transcriptase domains cluster into six distinct clades: *Ale*, *Tork*, *Ikeros*, *TAR*, *Ivana*, and *SIRE*. In contrast, the Ty3/*Gypsy* families are sorted into five clades: *Galadriel*, *Reina*, *CRM*, *Tekay*, and *Athila* (Llorens *et al.* 2009, Neumann *et al.* 2019). Advances in computational biology have further refined LTR-RT identification and classification through tools such as LTR retriever (Ou and Jiang 2018), LtrDetector (Valencia and Girgis 2019). These tools utilize signature-based methods, focusing on the structural features such as flanking LTR, rather than relying on known nucleotide sequences, to enhance accuracy.

While most existing software for predicting LTR-RTs provides fundamental categorization results, there’s an increasing need for more precise outcomes to facilitate detailed genome structure and functional analyses. Among these tools, LTR retriever stands out for its remarkable accuracy in LTR-RT identification, and its integration into the Extensive *de novo* TE Annotator (EDTA) pipeline has established it as the standard procedure for genome annotation (Ou *et al.* 2019). However, LTR retriever’s classification of LTR-RTs is limited to distinguish the Ty1/*Copia* and Ty3/*Gypsy* superfamilies. Despite being often regarded as inactive due to mutations, some TEs are actively expressed and play roles in cellular processes. Therefore, identifying their genomic locations is pivotal for phenotype studies. Telescope, a software developed by Bendall ML, addresses fragment assignment ambiguity by reassigning fragments based on a Bayesian model (Bendall *et al.* 2019). However, integrating the outputs of this software with the prediction results of established LTR-RTs involves complex procedures, which has hindered the broader adaptation and development of transposon quantification.

**Figure 1.**
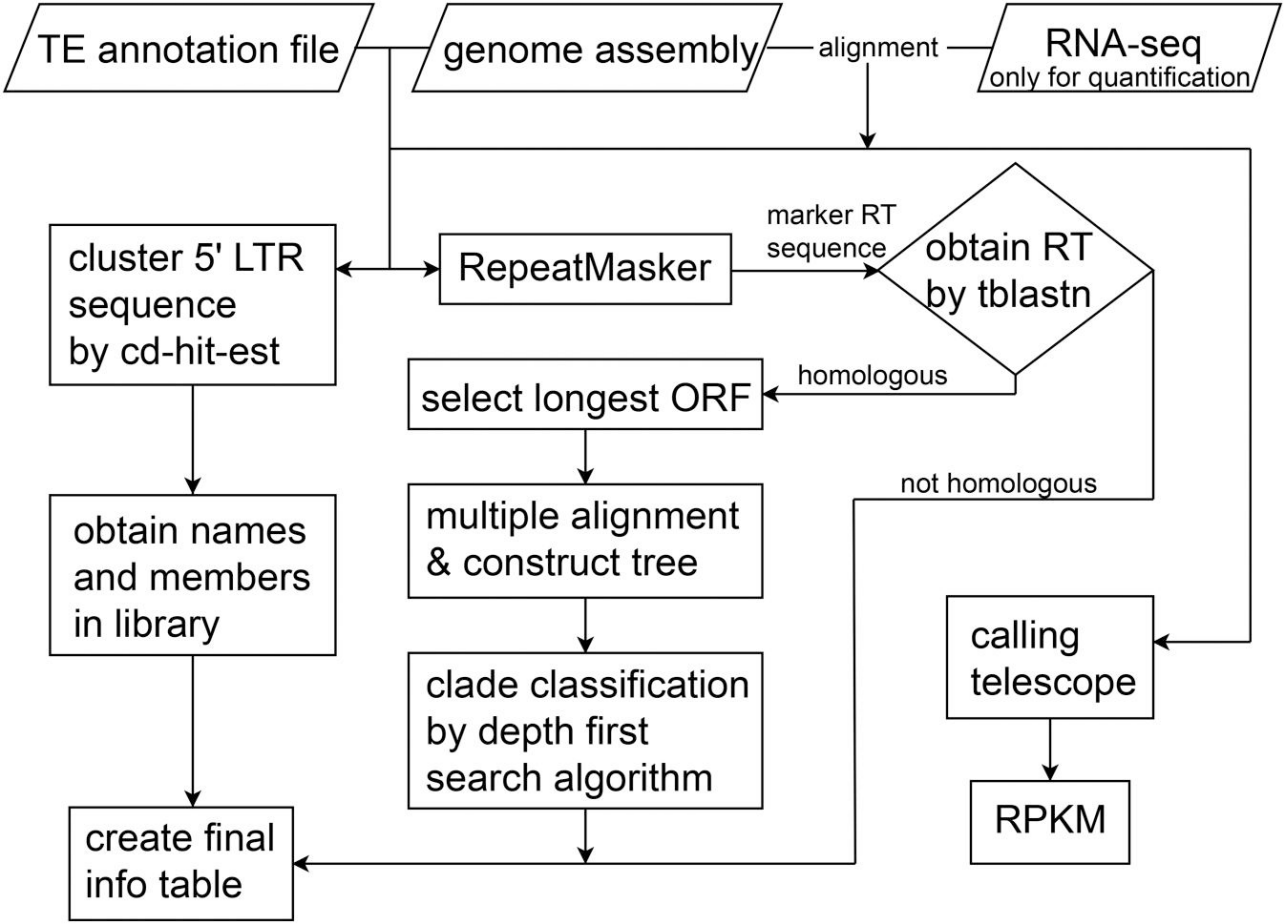
Standard flowchart for volcano software.

To address these challenges, we developed Volcano, a software that builds upon the LTR retriever results to enhance the precision in LTR-RT classification in plants (Fig. 1). Volcano estimates outbreak times and identifies the LTR-RT families that dominate the genome. With a user-friendly, one-click interface and accessible scripts, it streamlines the annotation process for users. Volcano uses an improved depth-first search algorithm to classify the different clades based on REXdb. Additionally, it integrates Telescope, simplifying the calculation of single-locus resolution for TE expression. Notably, Volcano is the first in its class to combine two functions for automatic and accurate quantification and classification. By refining LTR-RT annotation, this bioinformatics pipeline supports high-quality gene annotation and provides deeper insights into the effects of LTR-RTs on genomic architecture. Its quantitative analyses shed light on how TEs migrate within the genome and the host organism’s response, contributing a better understanding of genome structure and biodiversity.

## 2 Implementation

### 2.1 Classification *via* long terminal repeat

LTRs are conserved regions within LTR-RTs and they play a pivotal role in the initial familial identification. In this study, we introduce Volcano, a tool that utilizes summary tables from LTR retriever (specifically, the “pass.list” file) and the reference genome as its inputs. LTR-RT classification typically hinges on the LTR or the reverse transcriptase (RT) sequence. As a starting point, we extract the LTR sequences for subsequent classification.

Leveraging insights from previous studies (Muszewska *et al.* 2011, Liu *et al.* 2018), we use CD-HIT (Huang *et al.* 2010) to cluster similar sequences into groups with a minimum sequence identity of 80%. From these groups, consensus sequences are generated by the cd-hit-est software with parameters “-c 0.8 -aL 0.8” (Wicker *et al.* 2007, Huang *et al.* 2024). Next, we cluster the LTR sequences to create a comprehensive LTR library and list. To mask the genome, the RepeatMasker tool (Smit *et al.* 2015) is used, using the LTR sequences as the library. Finally, we ascertain the copy numbers and coverage for each repeat element family.

### 2.2 Classification of full-length LTR retrotransposons

The multitude of reverse transcriptase nestled within LTR-RTs can be further demarcated based on LTR-RT clades. To achieve this, we exploit LTR retriever or EDTA results to garner full-length LTR-RT sequences and assign distinct accessions to each LTR-RT. These accessions are then incorporated into the LTR-family list. Additionally, we include marker sequences from the study by Llorens *et al.* (2009), and leverage BLAST to obtain reverse transcriptase sequences. This process allows us to pinpoint the longest open reading frames (ORFs) for each LTR-RT. Through multiple sequence alignments, we construct *Copia* and *Gypsy* trees, inclusive of the marker reverse transcriptase sequences, and estimate distances. Subsequently, we apply domain-based classifications to the conjoined accession list along with the LTR-family data.

To study active LTR-RTs within each family, we compile a list of library names along with the corresponding numbers of activated LTR-RTs. This list is then appended to the coverage file. By reevaluating LTR-RT family types rooted in LTR sequences and conserved reverse transcriptase sequences, we finalize our classification. Lastly, we amalgamate the coverage file, family copy numbers, and tree ID data into a comprehensive, singular file.

### 2.3 Quantification and precise classification of LTR-RTs

For users with RNA sequence data, Volcano integrates with the Telescope software to calculate the single-locus resolution of transposable element expression (Bendall *et al.* 2019). To utilize this feature of this tool, users need to provide the “pass.list.gff3” file generated by LTR retriever, the reference genome, and a sorted SAM file aligning RNA-seq data to the reference genome.

The subsequent statistical report and the quantitative file encompass FPKM (fragments per kilobase of transcript per million mapped reads) values, enabling detailed analysis of LTR-RT expression patterns and their associations with genes. The amplification and expression of LTR-RTs add to genome complexity and, to a certain degree, function as a filter for genes. The Volcano software illuminates the ways in which LTRs contribute to species differentiation and adaptation.

### 2.4 Clade level classification according to an improved depth first search algorithm

We developed a Python script that interprets a Newick-formatted phylogenetic tree, collates paths proceeding from the root to labeled nodes, and categorizes each node based on the longest path overlapping with labeled nodes. Depth-first search (DFS) was prioritized over breadth-first search (BFS) for three key reasons: (i) DFS naturally preserves complete root-to-node paths during traversal, critical for overlap calculation; (ii) Stack-based DFS [O(h) space] outperforms BFS [O(w) space] for deep phylogenetic trees where tree height (h) much less than maximum width (w); (iii) Depth-prioritized exploration mirrors evolutionary divergence patterns. These processes use a depth-first search (DFS) algorithm (Tarjan 1972) tailored for this task and is executed in three stages. The first stage relies on the reverse transcriptase sequence, uses pattern matching to distinguish between tentative names and markers, and then builds a multiway tree based on its depth. The second stage involves traversing the multiway tree using the subsequence traversal algorithm to track the path of each marker in the tree. The final stage reuses the subsequent traversal algorithm again to navigate the tree, identify tentative node names, record the position paths of the nodes, and then determine the overlap of paths between the current node and the marker node, with the highest overlap being chosen as the category of the current node.

**Figure 2.**
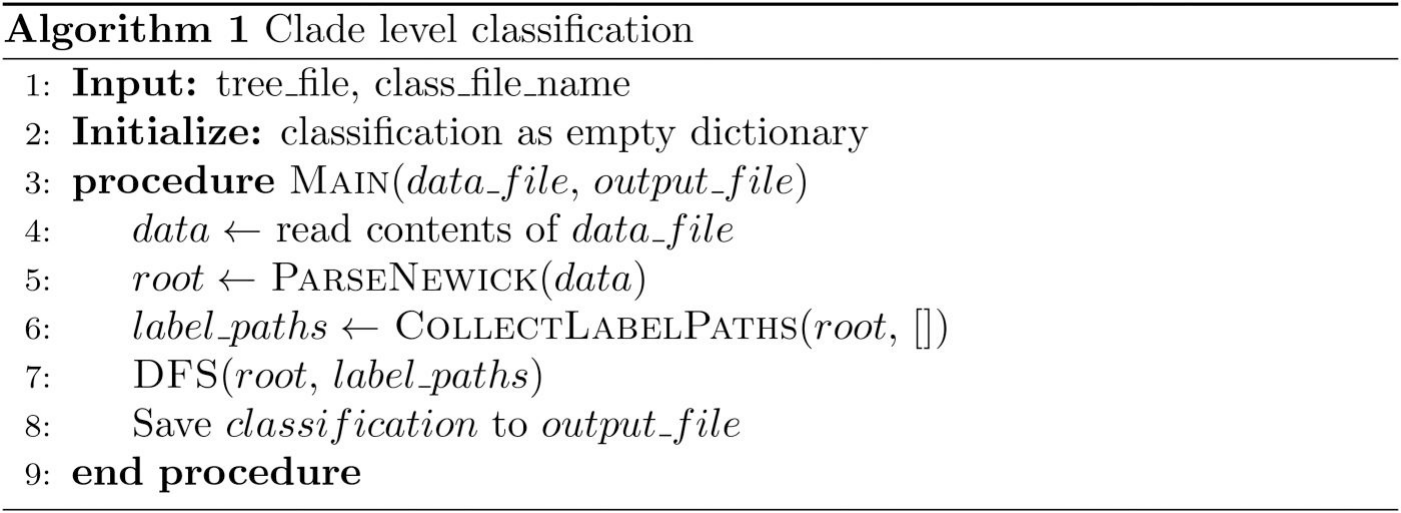
Pseudocode of simplified tree classification process.

In detail, the script creates a “TreeNode” class, processes input files, and uses regular expressions to match and extract node information. The “parse_newick” function constructs the tree, while the “collect_label_paths” function compiles paths leading to labeled nodes. The “classify_node” function calculates the overlap between paths and assigns classifications. The DFS function traverses the tree, appending nodes to the current path and classifying leaf nodes. The main function oversees the reading of the tree data, building the tree structure, gathering label paths, classifying nodes, and saving the results to a CSV file. Command-line arguments are used to dictate input and output file paths. Taken together, this script effectively unites tree parsing, DFS, and classification to offer a coherent and organized approach to phylogenetic analysis, while being mindful of performance and data integrity (Fig. 2).

After the categorization process, Volcano also provides an R script to visualize the tree using differing colors for various clades, and users can plot *copia* and *gypsy* trees based on the output files. All major scripts are explained on the wiki page (https://github.com/Suosihe/volcano_LTR/wiki). The functions and specific performance of each module in the program are described in the supplementary materials.

### 2.5 Illustrative examples

The expansion of the Asteraceae genome is predominantly due to repetitive sequences, which also play a significant role in its extraordinary adaptability (Reyes-Chin-Wo *et al.* 2017, Vitales *et al.* 2019). Utilizing high-quality genome assemblies of three Asteraceae species—*Chrysanthemum lavandulifolium*, *Carthamus tinctorius* and *Erigeron canadensis* (Hereafter abbreviated as CL, CT, and EC respectively)—we applied both EDTA and Volcano pipelines (Laforest *et al.* 2020, Nakano *et al.* 2021, Wu *et al.* 2021). Our aim was to discern patterns associated with long terminal repeat retrotransposons (LTR-RTs) within Asteraceae genomes.

**Figure 3.**
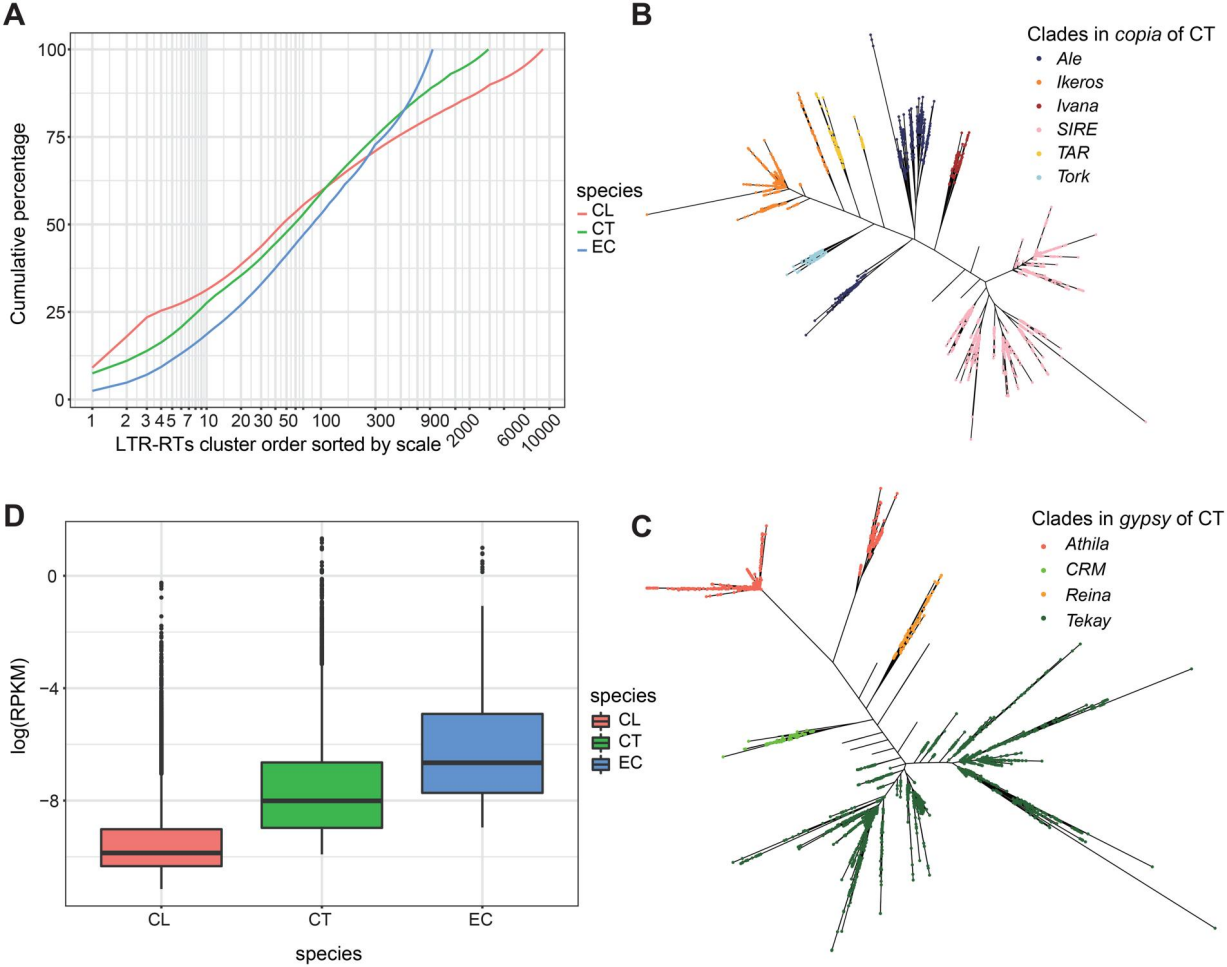
Long terminal repeat retrotransposons (LTR-RTs) of three Asteraceae genomes. (A) Cumulative frequency of LTR-RT clusters in the genome, sorted and ordered descending according to their coverage. The horizontal axis is log-transformed. Most of the LTR-RTs can be categorized in a few clusters, and there is basically only one LTR-RT in the later clusters. Phylogenetic trees of Ty1/*Copia* (B) and Ty3/*Gypsy* (C) retrotransposons are constructed from reverse transcriptase sequences in *Carthamus tinctorius* by mafft & fasttree and customized visualization scripts. Each colored circle represents a single intact LTR-RT. Different colored circles represent different clade. (D) Reads Per Kilobase per Million mapped reads (RPKM) of the expressed LTR-RTs. *Chrysanthemum lavandulifolium*, *Carthamus tinctorius* and *Erigeron canadensis* are abbreviated as CL, CT, and EC respectively.

The summative tables uncovered intact LTR-RTs complete with accurate coordinates and structural details. These LTR-RTs account for 66.9%, 53.16%, and 30.6% of the genome size (2.985 Gb, 1.057 Gb, and 426 Mb) in CL, CT, and EC, respectively. In particular, we pinpointed 56 965 intact LTR-RTs in the CL genome, 22 200 in the CT genome, and 2400 in the EC genome. Grouping full-length LTR-RTs using their 5’-LTR sequences resulted in 8732, 2919, and 951 clusters. Notably, the top 46, 58, and 84 clusters housed 50% of the intact LTR-RTs (Table 1, Fig. 3A).

**Table 1.** Volcano results of three Asteraceae species.

Species	*Chrysanthemum lavandulifolium*	*Carthamus tinctorius*	*Erigeron canadensis*
Genome size(bp)	2 985 010 845	1 057 104 314	426 326 274
Total LTR-RTs	2 172 947	517 129	117 666
Total LTR-RTs length (bp)	1 996 745 170	561 962 772	130 430 600
Total LTR-RTs percentage	66.9	53.16	30.6
Intact LTR-RTs	56 965	22 200	2400
Cluster number	8732	2919	951
Average number for each cluster	6.52	7.61	2.52
Expressed LTR-RTs	17 521	7131	914

At the clade level, the three species were classified. “Ale” in *copia* and “CRM” in *gypsy* constituted the majority. These details are outlined in Table 2, with visualization pertaining to CT provided in Fig. 3B and C.

**Table 2.** Clade classification of LTR-RTs in three Asteraceae species.^a^

type	clade	*Chrysanthemum lavandulifolium*	*Carthamus tinctorius*	*Erigeron canadensis*
Ty1/*copia*	*Ale*	1703	734	252
	*Ikeros*	907	1980	164
	*Ivana*	771	128	53
	*SIRE*	11 809	4350	118
	*TAR*	1309	152	22
	*Tork*	168	172	296
	unclassified	2185	1925	494
Ty3/*gypsy*	*Athila*	131	2767	199
	*CRM*	134	144	19
	*Galadriel*	64	0	0
	*Reina*	4897	112	51
	*Tekay*	1810	4898	124
	unclassified	8127	727	109

a These numbers of LTR-RTs are classified to different clade or unclassified.

Moreover, we quantified LTR-RT expression using publicly available RNA sequencing data for CL, CT, and EC genomes, contrasting them with their assembled genomic libraries. Notably, smaller genome sizes correlated with fewer detected or expressed LTR-RTs. Among the intact LTR-RTs in the three Asteraceae genomes, our quantitative analysis utilizing Volcano identified 17 521 expressed LTR-RTs in CL, 7131 in CT, and 914 in EC (Table 1, Fig. 3D).

## 3 Conclusion

We developed the Volcano software, a user-friendly tool that can be easily installed via Conda and runs exclusively on the widely supported Perl and R languages, eliminating the need for complex compilation. Users only need to provide FASTA files and outputs from commonly used tools such as EDTA or LTR retriever. Volcano integrates three external software packages to efficiently classify LTR-RTs using pre-established parameters. Importantly, the focus exclusively on full-length LTRs for classification significantly enhances processing speed. The software is designed to operate in both single-threaded and multi-threaded modes, offering flexibility to suit varying computational needs. Compared to its counterparts, volcano is comprehensive and capable of categorizing LTR-RT in detail (Table 3). It can improve the productivity in plants of bioinformaticians to a great extent.

**Table 3.** Comparison of volcano and similar software.

software	classify to subfamily	classify to clade	cluster LTR-RTs	quantification	visiualization
volcano	Y	Y	Y	Y	Y
EDTA	Y	N	N	N	N
LTR_retriever	Y	N	N	N	N
telescope	N	N	N	Y	N
inpactor2	Y	Y	N	N	N
TEsorter	Y	Y	N	N	Y

The results generated by our software provide users with a deeper understanding of LTR-RT classes and their expression patterns, fascinating detailed exploration of their genomic impact. Volcano constructs phylogenetic trees based on reverse transcriptase (RT) sequences for two LTR-RT subclasses: Ty3/*gypsy* and Ty1/*copia*. An enhanced depth-first search algorithm is used to classify the different clades based on the REXdb. By leveraging marker sequences and tree annotation files, users can efficiently delve into clade-level classification. In addition, the built-in quantification script allows for precise quantification of LTR-RTs expression, a capability not available in existing tools. This methodology has demonstrated its effectiveness in genome assemblies for species such as chicory and *Scaevola taccada* (Shen *et al.* 2023a,b). Considering that the rich diversity of eukaryotic organisms is also reflected in LTR-RTs, and that the types of LTR-RTs in fungi and animals are very different from those in plants, our volcano software is currently designed specifically for LTR-RTs of plant genomes only. We are confident that Volcano provides reliable classification and quantification of this critical genomic component, enabling users to explore genomic results more thoroughly in plants.

## Supplementary Material

vbaf162_Supplementary_Data

## Data Availability

Data available on request.
