# Supplementary material for: Volcano: a pipeline to characterize long terminal repeat-retrotransposons families in plants
Source: Bioinform Adv. 2025 Jul 4;5(1):vbaf162. doi: 10.1093/bioadv/vbaf162 (PMC12349922; doi:10.1093/bioadv/vbaf162)
Supplement: vbaf162_Supplementary_Data [file vbaf162_supplementary_data.docx]

SUPPLEMENTARY MATERIALS

**Detailed Description of the VOLCANO Pipeline**

**Initial Input Processing**

As Extensive *de novo* TE Annotator (EDTA) (Ou *et al*., 2019) and LTR_retriever (Ou and Jiang, 2018) remain the gold-standard tools for repetitive element annotation in genomics, despite their limited LTR-RTs classification resolution (only distinguishing between *copia* and *gypsy* superfamilies), we utilized their standard output file ‘prefix.pass.list’ as the input for our refined classification pipeline. This file format was selected due to its widespread availability and compatibility with downstream analyses.

**LTR Extraction and Preprocessing**

Our pipeline (list_to_5ltr.pl) first extracts flanking LTR sequences from LTR-RTs. These sequences were then clustered using cd-hit-est (Huang *et al.*, 2010) under the "80%-80%-80%" rule (80% sequence identity over 80% of the length for 80% of sequences) (Wicker *et al.*, 2007) with default parameters ‘-c 0.8 -aL 0.8 -T 0 -M 0 -n 5 -d 200’. Users can set different ones in the main script, volcano.sh. Subsequently, RepeatMasker was employed with parameters ‘-pa 80 -q -no_is -norna -nolow -div 40 -cutoff 225’ (along with our custom scripts (obtain_lib_list.pl and fam_coverage.pl) to mask LTR sequences in the genome (parallel number and masking percentage diverged from consensus are adjustable for the user), while simultaneously calculating copy numbers and coverage rates for each repetitive element family.

**Reverse Transcriptase Sequence Processing and Quality Control**

Using the masked genome library, we extracted reverse transcriptase sequences through our list2_ltr_seq.pl script. These sequences underwent rigorous quality filtering *via* tBLASTn comparison against experimentally verified marker sequences (parameters: ‘-max_target_seqs 1000000000 -max_hsps 1 -evalue 10e-5’). Low-confidence LTR-RT sequences (failing to meet confidence thresholds) were annotated with NULL values in the classification field.

**Phylogenetic Analysis and Classification**

Filtered reverse transcriptase sequences were aligned using MAFFT (default parameters, ‘--op 1.53 --ep 0.0 --maxiterate 0 --quiet --thread 1 ) (Katoh *et al.*, 2002, 2013) which combined progressive methods (FFT‐NS‐2) and iterative refinement (FFT‐NS‐i) and phylogenetic trees were constructed rapidly with FastTree (default parameters) (Price *et al.*, 2010) combined a maximum likelihood framework with heuristic optimizations (Subtree Pruning and Regrafting and Nearest Neighbor Interchanges) and approximate models (CAT instead of full Gamma for site rate heterogeneity). Our improved depth-first search algorithm (implemented in classify_clade.py, explained in detail in the main text) identified the nearest marker sequence for each reverse transcriptase, followed by comparison with both marker sequences and clade reference tables to determine clade membership. This process generated comprehensive classification tables.

**Data Integration**

A series of custom Perl scripts (assign_domain_based.pl, obtain_lib_list_num.pl, re_judge2.pl, and add_family_info.pl) were developed to integrate classification results, copy numbers, coverage rates, and other metrics into a unified output file.

**Visualization Module**

For visualization, our clade_plot.R script generates publication-quality graphics by calling R packages such as ‘ggtree’ and ‘treeio’ (Wang *et al*., 2020; Yu *et al*., 2017), requiring the phylogenetic tree of reverse transcriptase sequences and their corresponding clade-level classification file as inputs. The script performs coloring of LTR-RTs according to different clades.

**Expression Analysis**

The tel.sh workflow standardizes output ‘prefix.pass.list.gff’ from either EDTA or LTR_retriever, processes it through Telescope (default parameters) (Bendall *et al.* 2019) via a Bayesian statistical model and an expectation-maximization (EM) algorithm for TE expression quantification, and finally calculates LTR-RT expression levels using our RPKM.R script, providing normalized expression values in Reads Per Kilobase per Million mapped reads (RPKM) units.

**Computational Efficiency Benchmarking**
Volcano's resource efficiency was evaluated on three plant genomes spanning 400 Mb to 3 Gb. Tests were conducted on a Linux server (10-core CPU, 24 GB RAM) under typical workload conditions. As shown in below, runtime scales sublinearly with genome size due to parallel processing optimizations:

- **400 Mb genome**: 30 min (peak RAM: 8.2 GB)
- **1 Gb genome**: 87 min (peak RAM: 14.1 GB)
- **3 Gb genome**: 176 min (peak RAM: 21.3 GB)
  *The observed memory footprint remained within affordable ranges for institutional computing clusters, demonstrating practical feasibility for large-scale phylogenetic analyses.*

**Reference**

Bendall,M.L. *et al.* (2019) Telescope: Characterization of the retrotranscriptome by accurate estimation of transposable element expression. *PLoS Comput Biol*, 15, e1006453.

Huang, G., Bao, Z., Feng, L. *et al.* (2024) A telomere-to-telomere cotton genome assembly reveals centromere evolution and a Mutator transposon-linked module regulating embryo development. *Nat Genet*, 56, 1953–1963.

Katoh, Misawa, Kuma, Miyata (2002) MAFFT: a novel method for rapid multiple sequence alignment based on fast Fourier transform. *Nucleic Acids Res*, 30:3059-3066.

Katoh, Standley (2013) MAFFT multiple sequence alignment software version 7: improvements in performance and usability. *Molecular Biology and Evolution*, 30:772-780.

Ou,S. *et al.* (2019) Benchmarking transposable element annotation methods for creation of a streamlined, comprehensive pipeline. *Genome Biol*, 20.

Ou,S. and Jiang,N. (2018) LTR_retriever: A highly accurate and sensitive program for identification of long terminal repeat retrotransposons. *Plant Physiol*, 176, 1410–1422.

Price, M.N., Dehal, P.S., and Arkin, A.P. (2010) FastTree 2 -- Approximately Maximum-Likelihood Trees for Large Alignments. PLoS ONE, 5(3):e9490.

Wang, L. G., Lam, T. T. Y., Xu, S., Dai, Z., Zhou, L., Feng, T., ... & Yu, G. (2020). Treeio: an R package for phylogenetic tree input and output with richly annotated and associated data. *Molecular biology and evolution*, *37*(2), 599-603.

Wicker,T. *et al.* (2007) A unified classification system for eukaryotic transposable elements. *Nat Rev Genet*, 8, 973–982.

Yu, G., Smith, D. K., Zhu, H., Guan, Y., & Lam, T. T. Y. (2017). ggtree: an R package for visualization and annotation of phylogenetic trees with their covariates and other associated data. *Methods in Ecology and Evolution*, *8*(1), 28-36.
